# Modelling resilience in adolescence and adversity: a novel framework to inform research and practice

**DOI:** 10.1038/s41398-019-0651-y

**Published:** 2019-11-26

**Authors:** Gin S. Malhi, Pritha Das, Erica Bell, Greg Mattingly, Zola Mannie

**Affiliations:** 1 Academic Department of Psychiatry, Northern Sydney Local Health District, St Leonards, NSW Australia; 2 Sydney Medical School Northern, University of Sydney, Sydney, NSW Australia; 3 CADE Clinic, Royal North Shore Hospital, Northern Sydney Local Health District, St Leonards, NSW Australia; 4 Washington University School of Medicine, St. Louis, MO 63110 USA; 5 NSW Health and Royal North Shore Hospital, Northern Sydney Local Health District, St Leonards, NSW Australia

**Keywords:** Psychiatric disorders, Human behaviour

## Abstract

Recent conceptualisations of resilience have advanced the notion that it is a dynamic and multifaceted construct. However, its adaptive components, especially those forged by adversity, have not been fully realised, and its neurobiological and psychosocial underpinnings are yet to be meaningfully integrated. In part, this is because a developmental perspective is often neglected in the formulation of resilience. In this review, we consider the findings of resilience research, with a specific emphasis on the developmental period of adolescence. To bridge the gaps in our current understanding, we propose a model of resilience that is predicated on experiencing adversity. Specifically, our model provides a sophisticated insight into the components of resilience, which, together with intrinsic features, involves facilitation of, and skill acquisition via strengthening processes we term tempering and fortification. The model also points to the potential trajectories of adversity-driven resilience and forms the basis of a framework that allows for individual variance in resilience, and the identification of both neurobiological and psychosocial targets for prevention and therapeutic interventions.

## Introduction

Stress is associated with an increased incidence of mental and physical health problems and decreased well-being. But remarkably, not all individuals who have been exposed to toxic stress or adversity develop these problems, even when their adverse experiences are severe and protracted. In fact, recent evidence suggests that up to two-thirds (65.7%) of individuals undergoing adversity remain relatively unscathed^1^—exhibiting what is generally referred to as ‘resilience’.

In recent years, the conceptualisation of resilience has undergone a paradigm shift, and is increasingly viewed as a dynamic process, akin to the acquisition of a skill, as opposed to a fixed characteristic^2^. As such, resilience is thought to be determined by both extrinsic (environmental) and intrinsic (genes and personality) factors^3^ and the interactions between the environment and genetic variants^4^. The most significant environmental factor that is an essential prerequisite for the development of resilience is adversity, and in this context, resilience is conceptualised as a positive adaptation, or ‘competence’^4–6^. Thus, resilience is increasingly regarded as a sophisticated and multifactorial construct^7^ with both neurobiological and psychosocial underpinnings, that is tangible across emotional, cognitive, behavioural, social and psychological domains of functioning^8^. Therefore, in broad terms, we define resilience as adaptive functioning after adversity, and this functioning can span identified multiple domains. However, its underlying mechanisms, and how it emerges from adversity, remain poorly understood^9–14^.

A key modelling limitation of resilience has been the failure to incorporate a developmental perspective and in particular, one that maps its formation during adolescence. Therefore, this review (1) conceptualises resilience as being primarily shaped by adversity, (2) emphasises the pivotal role of adolescence in its development and (3) presents a novel multifaceted framework that models its emergence from adversity during adolescence and incorporates formative neurobiological and psychosocial factors. The model and framework provide a basis upon which to build a program of research that has salience for clinical practice.

## Reconceptualization of resilience

Current understanding no longer regards resilience as an individual trait^2,15,16^, although some continue to consider it a useful concept^17,18^. Resilience has also been conceptualised as the absence of psychopathology^5,19^, but this perspective has been critiqued for its narrow focus, because even people with significant psychopathology can function well and be resilient. Furthermore, even those without psychopathology are not necessarily resilient. Indeed, it has been suggested that both resilient and vulnerable phenotypes can co-present within the same disorder^20^.

This has prompted the reconceptualization of resilience so that it better reflects its relationship with adversity. It is important to note that in *this* context, adversity refers to threat- or deprivation-related forms of stress exposure^21^. This paradigm-shift in how resilience is regarded, from a limited or specific trait that is independent of psychopathology, to a more sophisticated adversity-predicated construct, warrants examination and further elucidation so as to advance a deeper understanding of the mechanisms driving its development.

There are two principal intrinsic factors that drive the conception of resilience as a trait, namely, its association with personality^22,23^ and supposed derivation from genetic factors^5,24^. Together, these are thought to inform individual stress response variability and determine a trajectory either towards the development of maladaptive behaviours and increasing vulnerability to the emergence of psychopathology, or towards the development of resilience and adaptation. With respect to personality, positive associations between trait resilience and extraversion, openness to experience, agreeableness and conscientiousness and negative associations with neuroticism have been reported in adults^16,17,23^. However, the few studies that have been conducted in adolescents reveal inconsistent associations^25–27^. Indeed, among Japanese adolescents, neuroticism was predictive of resilience only among females and extraversion only among males^25^, and in a study of Italian adolescents another personality factor, dispositional optimism, was positively associated with resilience^28^. However, and crucially, in all of these studies, resilience is assessed outside the context of adversity and therefore, this research does not necessarily inform us as to how resilience emerges in response to adversity.

**Fig. 1:**
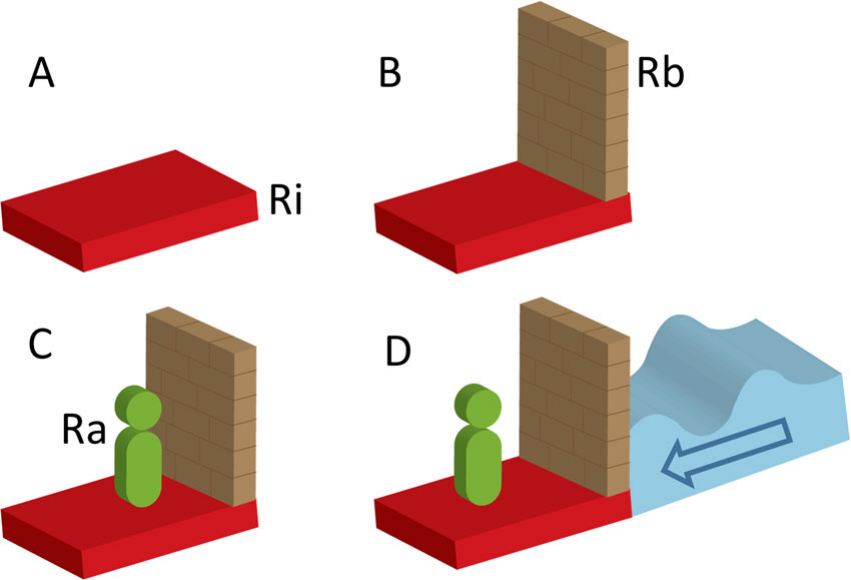
This schematic shows components involved in adaptive resilience. Depicting this concretely, resilience is shown as having four components, **a**–**d**. **a** The base (in red) comprises of intrinsic (*R*_i_) factors established from birth, e.g. genetics whose expression can be modified by experience through epigenetic mechanisms and personality factors. It represents the foundation upon which further resilience (the wall) is constructed. **b** On top of and emanating from intrinsic factors are the ‘structures’ (depicted by the wall) that represent the neurobiological and psychosocial ‘constructs’ through which resilience is built (*R*_b_). These constructs and component factors are varied and interconnected (see underlying mechanisms section). **c** A supportive environment facilitates the processing and regulation of neurobiological and psychosocial factors that builds and promotes adaptive resilience (*R*_a_). Therefore, the green figure represents an individual’s ability to respond and adapt, as well as address the changing needs of *R*_i_ and *R*_b_. **d** Complete resilience can only be achieved in response to adversity (depicted by the pressure exerted by the water on the wall). The level of the water and the amplitude of its waveform represents the magnitude of adversity.

The second source of intrinsic resilience is the genetic makeup of the individual. Genetic polymorphisms associated with vulnerability to adverse environments may also be associated with resilience or responsivity to positive environments^29^. The *genetic differential susceptibility* hypothesis suggests that a select number of polymorphisms, previously considered as ‘risk’ genes in the diathesis-stress model, are better regarded as ‘plasticity’ genes^30^ that are inherently ‘neither good nor bad’. Therefore, specific genetic variants and their expression may be the main determinants of susceptibility or resilience in the context of adversity. Specifically, the hypothesis posed by *genetic differential susceptibility* is that some individuals are more susceptible to environmental influences, resulting in either worse or better outcomes^31^. Additionally, individual experience may modify gene expression via epigenetic mechanisms to influence optimisation of individual adaptation as a basis of resilience^20,32–35^. Therefore, both *personality traits* and *genetic differential susceptibility* can be regarded as core *intrinsic* features of resilience, (denoted as *R*_i_ in Fig. 1) that form the basis for individual variation in future adversity predicated resilience.

## Features of adversity

The dynamic nature of resilience is influenced by the type, timing, intensity and duration of adversity, that creates variability in when, and how, it emerges, as well as what shape it assumes. The most researched types of adversity are childhood trauma exposure and socioeconomic adversity. These can be assessed in adulthood retrospectively but are best assessed in childhood. Exposure to childhood trauma (physical, sexual and emotional abuse and physical and emotional neglect) has been shown to reduce resilience as indexed by active problem solving abilities in a representative community sample spanning mid-adolescence (14 years) to old age (92 years)^36^. In this same study, resilient coping was associated with reduced distress and found to mediate the relationship between adversity and distress. Furthermore, in a community sample of young adults (aged 18–35 years), resilience as measured by the Brief Resilience Scale (BRS^37^), a measure of the ability to ‘bounce back’ from verbal abuse/threat, sexual abuse and physical neglect as well as emotional support (an indicator of resilience) predicted an attenuation in prodromal psychotic symptoms^38^. And finally, in a sample of adult primary care patients, resilience, as measured by the Connor Davidson-Resilience Scale (CD-RISC)^39^, partially moderated the effect of childhood emotional, physical and sexual abuse, physical and emotional neglect as well as household dysfunction on anxiety levels^40^ and depression^41^ by attenuating emotion dysregulation. Resilient functioning, as indexed by greater spirituality, higher emotional intelligence and supportive friendships on the CD-RISC, is effective in diminishing the negative impact of victimisation in childhood, as well as depression and anxiety in young adulthood^42^.

This evidence from a diverse range of studies indicates that there are many specific kinds of adversities that independently or jointly produce resilience, but all are commonly either threat- or deprivation-related^21^. This is important because different types of adversity, differing degrees of intensity and duration produce a variable allostatic load on stress response systems. This variability in the exerted ‘pressure’ necessitates corresponding changes in resilience so as to proportionally counter their impact. Allostatic load refers to the cumulative physiological cost (‘wear and tear’) that results from adaptive shifts made across multiple systems to match internal functioning to external or environmental demands both within the brain, and stress systems, that are at the core of this adaptation^43^. For example, there is evidence that specific subtypes of childhood adversity mechanistically produce differential dysregulation of the hypothalamic–pituitary–adrenal (HPA) axis and inflammatory system^44^, and specific genes may be independently or collectively associated with physical abuse or physical neglect^34,35,45^. Specifically, physical abuse has been shown to exaggerate HPA axis and inflammatory responses, whereas emotional abuse is thought to delay physiological recovery from stress and, in doing so, prolongs exposure to glucocorticoids. Similarly, low socioeconomic status (SES) frequently activates physiological stress systems and increases both cortisol and inflammation^44^. Furthermore, physical abuse and physical neglect, although they have some genes in common, are also thought to be independently associated with variation in specific genes^45^. This means that the *type* of adversity experienced may play a key role in determining which neurobiological systems are impacted and in turn, via which processes resilience is most likely to take form.

**Fig. 2:**
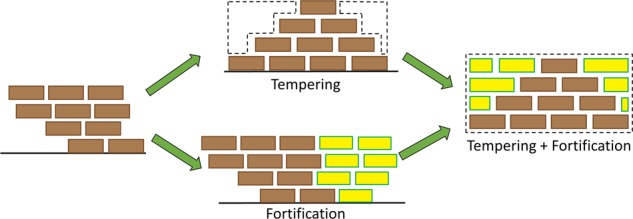
This schematic shows how tempering and fortification stabilise structures (representing neurobiological and psychosocial processes) that were destabilised by adversity. Tempering involves redistribution of pre-existing resources to enhance functioning, but this redistribution may create brittleness or weaknesses in some structures while strengthening others within stress-responsive systems. This may, on occasion, inadvertently destabilise structures. To strengthen these functions and eliminate or diminish weaknesses, acquisition and addition of new resources is required, through a fortification process.

Hence, the evolution of resilience is perhaps differentially initiated based on the unique combination of intrinsic resilience (denoted as *R*_i_ in Fig. 1) and an additional reconstruction of stress-responsive systems, referred to as resilience building (denoted as *R*_b_ in Fig. 1). In a healthy environment, resilience building emerges from exposure to enriching environments or positive life experiences. Animal evidence has revealed that natural-enriched environments and optimal early-life experiences build resilience via neurobiological changes^46,47^. Indeed, in humans, healthy lifestyles (healthy diet, adequate physical activity and sleep) and supportive environments that provide a sense of security and belonging, self-worth, realistic mastery and control from an early age, all contribute to building and promoting resilience^4,48^, through neurobiological and psychosocial mechanisms (see Underlying Mechanisms section below). Furthermore, mild and positive stressors may not destabilise the stress-responsive systems to a degree that demands resilience, in the first place. However, when allostatic load increases in response to adversity, the stress-responsive systems struggle to cope with the increasing pressure exerted on them, despite pre-existing intrinsic and built resilience. It is at this juncture that the dynamism of resilience as predicated by adversity is most evident, as allostatic load increases, and intrinsic and built resilience prove to be insufficient to cope with the pressure exerted by allostatic load and are eventually overcome. In this manner, allostatic load can bring about variable impairment in stress-response systems ranging from minimal and moderate to severe. This is when strengthening is needed to produce adaptive resilience (denoted as *R*_a_ in Fig. 1). This strengthening involves the use of *tempering* and *fortification* strategies that operate in tandem to repair or modify failing, compromised or impaired systems while strengthening existing stress-responsive systems (see Fig. 2).

## Tempering

Tempering is one process by which strengthening of systems enhances resilience. Tempering has many definitions, but the most precise definition for the processes we deem to be involved in resilience is “to make stronger and more resilient through hardship” (see Merriam-Webster dictionary). Because of the lack of a precise term for the processes involved in resilience in our field, we have had to borrow this term from other fields where it is used to strengthen and increase durability (e.g. alloy, glass and chocolate). In our clinical context, it is used in abstract and direct terms. It involves the engagement of skills that have been previously acquired through childhood experiences^49,50^, but perhaps through a lack of use, have remained underdeveloped by the time adolescence is reached (see Fig. 2). Activated by adversity, tempering involves the re-engagement and refinement of these dormant skills that are then reused to both repair and strengthen stress-responsive systems and optimise their functioning. However, as tempering realigns various skills and abilities and synchronises processes (producing immediate strength), it can simultaneously introduce common fragilities resulting in a degree of ‘brittleness’ and inflexibility within resilience.

## Fortification

Another means of strengthening resilience is fortification. Like tempering, ‘fortification’ is a process that has been used in other fields, e.g. nutrition sciences and the military but has been borrowed because it aptly explains the processes involved in the acquisition of resilience. Besides building defences against potential and actual damage or deficits, it also reduces or counters brittleness in structures to increase their stability. It repairs and modifies components of resilience that are imbalanced, compromised or impaired, and in effect bolsters any brittleness that may have been produced by tempering. Fortification achieves this by cultivating *additional new skills*. This means that new skills have to be learnt and acquired and this is particularly useful when allostatic load from adversity is excessive and significantly burdens stress-response systems. It’s important to note that there is probably also a temporal distinction between tempering and fortification and some key limiting factors that differentiate the two processes. For example, fortification is reliant on the acquisition of ‘new material’ and this takes time. If it is not possible to acquire these new skills, then fortification cannot be effectively deployed. Tempering on the other hand does not require new raw material but it does require the correct sort of ‘pressure’ to be applied at the right time and so it too may have a narrow window in which it occurs. Thus, tempering is likely to be more immediate but not as long lasting, unless the changes it produces can be built upon, perhaps through fortification. Therefore, tempering and fortification are iterative to some extent and may operate in a coordinated and seamless manner, but each of them can also occur independently (see Fig. 2).

## Impact of adversity

**Fig. 3:**
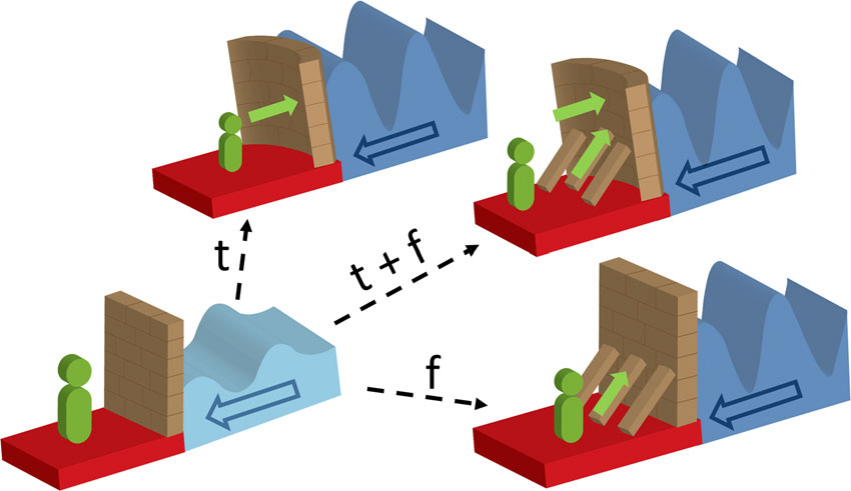
This schematic diagram illustrates how tempering and fortification strategies operate to enhance resilience in response to adversity. The ability to withstand increasing pressure induced by allostatic load (represented by the dark blue arrow) requires active engagement (represented by green arrow) to either reuse dormant but pre-existing skills (tempering (*t*)) and transfer or deploy such skills for use in new contexts (depicted by curvature in the tempered wall) to produce tempered resilience, or acquire additional skills (fortification (*f*); depicted by braced wall) to ‘further build’ resilience culminating in fortified resilience. To enhance stability, the tempered wall also needs fortification (depicted by the tempered and fortified wall (*t* + *f*) that represents tempered and fortified resilience). Tempering and fortification ultimately repair, modify and strengthen the integrity of stress-responsive systems (neurobiological and psychosocial), to produce adaptive resilience (*R*_a_). However, fortification may operate independently, when the systems have been overwhelmed. The intensity of adversity is depicted by the colour change from a lighter (low intensity) to a darker (high intensity) blue colour. The duration is depicted by the wavelength, which can be intermittent, short or prolonged. It is important to capture this variability in adversity as it reflects the varying degrees of allostatic load.

For those with minimal impairment of their stress-responsive systems, previously learned tempering strategies may be sufficient for them to maintain resilience, despite adversity. However, moderate impairment may give way to subthreshold symptoms or maladaptive behaviours, and these individuals may eventually require additional strategies that need to be learned and acquired to enact tempered and fortified resilience (denoted as *t* + *f* in Fig. 3).

Indeed, a focus on shared neurobiological and related psychosocial consequences of adversity, regardless of its features (type, intensity and duration), is an optimal strategy to demonstrate both its dynamism and multifaceted nature^32^. For instance, early life exposure to intermittently (duration) mild to moderate (intensity) controllable, but challenging, stress may induce *stress inoculation* to strengthen existing structures and this promotes resilience across multiple domains of adaptive functioning after adversity^14,51–54^. Based on animal evidence, stress inoculation emerges as experience- or learning-dependent ‘*vaccination*’ of stress systems and their associated emotion, cognitive and social processes and neural networks that subserve these processes^55–57^. This means that both exposure to high stress may fragment resilience while sheltering from adversity may cause weakened development^54^. Moderate exposure, possibly through trial and error, provides an opportunity to experience and practice the control and mastery of stress-responsive systems. However, inoculation is only workable up to a mild to moderate stress threshold. Beyond this, alternative strategies are needed.

When adversity exposure and associated allostatic load are excessive or chronic, they can produce variable impairment to the stress-responsive systems. This is when additional resources are required to counter the negative effects of adversity, but also repair any impairment, and modify failing systems while strengthening existing stress-responsive systems. Thus repair, modification and strengthening facilitates resilience through processes we refer to as *tempering* and *fortification* (denoted as *t* and *f*, respectively in Fig. 3). Note that together, tempering and fortification *(t* + *f)* produce adaptive resilience (*R*_a_).

**Fig. 4:**
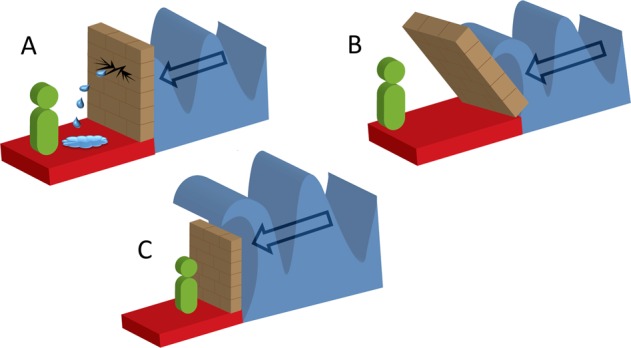
This schematic diagram shows the dynamic nature of resilience induced by the varied impact of allostatic load on the neurobiological and psychosocial structures depicted by the wall. When load is significant, it may compromise the integrity of the wall (representing neurobiological and psychosocial indices of resilience), to produce structural and associated functional changes. These changes are depicted by a breach in the wall (cracking and leakage in **a**) and excessive pressure (leaning of the wall in **b**). Alternatively, allostatic load may be too overwhelming for the stress-responsive systems to even begin to contend with (allostatic load flows over the wall in **c**). The changes can create variable instability in stress-responsive systems that can manifest as maladjusted behaviours, subthrehold symptomatology or psychopathology.

Notably, the adaptive acquisition component of resilience is especially important to those with underlying impairment or psychopathology. However, in such instances, the mechanisms driving its acquisition may vary as shown in Fig. 4.

## Timing of adversity exposure

Although there is mounting knowledge concerning the effects of adversity on the genetics, epigenetics and neurobiology of adults, little is known about these effects in adolescents^58^, where the focus has largely been on psychosocial determinants. Indeed, adversity-driven resilience is mostly examined in adulthood, where the deleterious effects of childhood adversity exposure have been fully realised and are perhaps easier to measure having achieved stability. This is a significant gap in our knowledge, because the adversity recounted in adulthood has usually been experienced many years prior and therefore, how this has shaped resilience at the time cannot be elucidated by assessing factors many years later. Indeed, the World Health Organisation (WHO) acknowledges that resilience results from developmental processes that can be strengthened over time, depending on the circumstances^59^. Therefore, examining resilience in adolescence and, in particular, the specific role of adversity is essential.

On the other hand, adolescence is a transitional period characterised by significant neurobiological and psychosocial changes in the context of expanding environmental demands and heightened sensitivity to social contexts^60^. These dramatic biopsychosocial changes and their neural underpinnings affect all domains of life; social, emotional and cognitive, along with physical maturation and consolidation of personality. This rapidly changing and turbulent environment poses significant challenges to examining resilience as compared to its study in adults. At the same time, these biopsychosocial changes may reflect or contribute to an increase in allostatic load that impacts resilience, and these are equally important to understand. Additionally, it is postulated that by disrupting normal brain development and heightening sensitivity to stress and adversity, adolescence rather than childhood adversity produces consistently potent negative effects that may lead to pervasive and enduring maladjustment^61–63^. Furthermore, despite the lifelong capacity of the brain to rewire in response to experiential learning, it is during adolescence that significant neuroplasticity occurs^64^, with the greatest effects on epigenetic modifications^33^. Simultaneously, adversity significantly increases during adolescence^65^, as individuation occurs and peer relationships (positive and negative) become increasingly important as opposed to those within the family. At the same time, major changes occur in stress-responsive systems such as HPA axis functioning^44^. This means that, with an expanding social network, the sources of adversity become more widespread, while resources required to cope with current or potential future adversity remain limited. Added to this, adolescents are yet to acquire experience in developing adaptive coping strategies to facilitate independence^66^. It has also been suggested that moderate stress during adolescence inoculates against later adversity^52^, through the acquisition of adaptive stress responses^32^.

Hence, as the threat and deprivation-related adversities occur at increased rates during adolescence^21^, a concerted effort to overcome these difficulties is crucial for well-being and general functioning.

### Adolescence as an important developmental stage for the emergence of long-lasting resilience

**Fig. 5:**
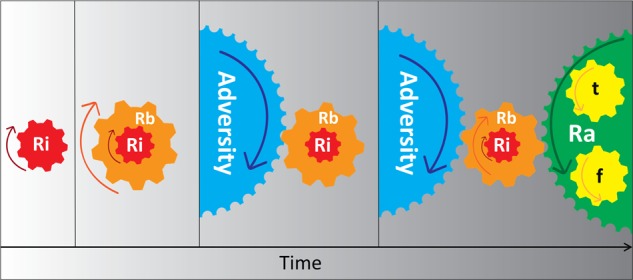
This schematic shows the intricate interdependence of the components of resilience with the additional tempering and fortification processes instigated by adversity. With the advent of adversity, the intrinsic and built components of resilience (*R*_i_ and *R*_b_) are engaged, but, depending on adversity features, may not be sufficient to maintain resilience. It is when adversity increases in intensity and subsequent allostatic load that new skills are acquired, and previously acquired skills are adapted and deployed for use in novel contexts to produce adaptive resilience *R*_a_, which acts to counter the effects of adversity on intrinsic and built resilience (*R*_i_ and *R*_b_). Therefore, tempering (*t*) and fortification (*f*) processes work in an integrated manner, to achieve adaptive resilience (*R*_a_).

Once resilience has been achieved, there need to be strategies in place to ensure that it is maintained and sustained over time. Therefore, at its core, resilience can be regarded as the adaptation of neural systems and their elaborated physiological, cognitive and behavioural manifestations in response to allostatic load^67^, and this requires all of its components to be engaged (see Fig. 5). It is in this context that adolescence provides an ideal opportunity to examine the development of resilience in terms of neural adaptation and behavioural change. The brain is highly ‘plastic’ during adolescence and is undergoing extensive reorganisation^68,69^, which makes it highly susceptible to the harmful effects of stress or adversity, but it is also at its most malleable and receptive to both *positive* (resilience-enhancing) and *negative* (vulnerability-inducing) influences. These factors make adolescence the ideal period for therapeutic and resilience-enhancing interventions. However, some adolescents with significant impairment may not readily demonstrate resilience because it is masked by excessive allostatic load created by extreme adversity that simply overwhelms all stress-responsive systems and this makes recovery, as an indicator of resilience, elusive.

## Underlying mechanisms linking neurobiology and psychosocial factors implicated in resilience

**Fig. 6:**
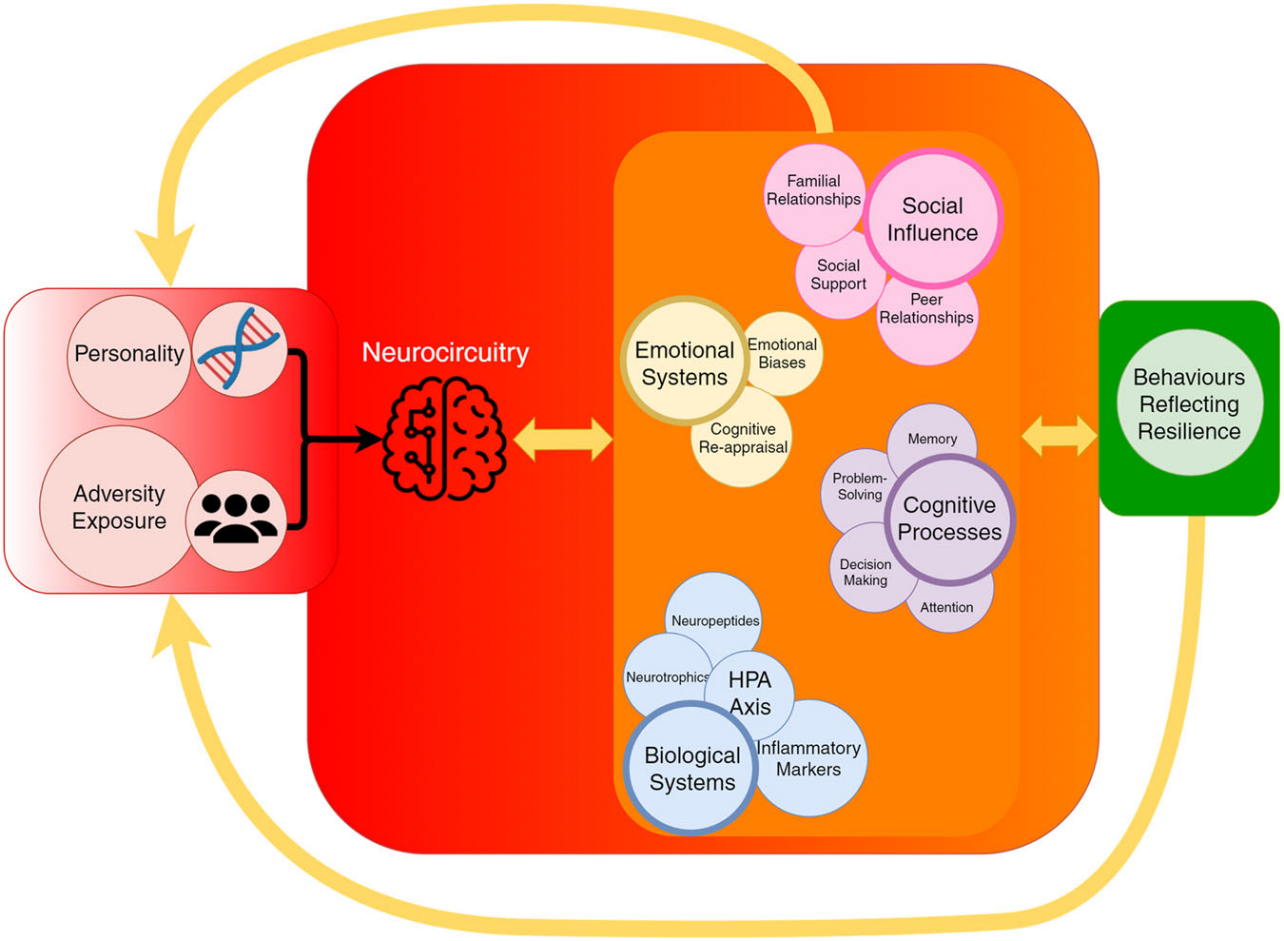
Schematic diagram of the neurobiological and psychosocial determinants of resilience that influence each other to produce adaptive behaviours reflecting adaptive resilience following adversity. When genes and personality, as intrinsic components of resilience, interact with varying degrees of environmental adversity, the individual’s stress-responsive neurocircuitry is activated and subsequent biological, emotion-cognitive and social structures are mobilised by individual intention, skill transfer and acquisition so they can be tempered and fortified. It is through these processes that resilience is achieved to ultimately manifest as adaptive behaviours. The latter in turn feeds back the experience to (i) the intrinsic factors to facilitate epigenetics and modify personality factors and (ii) the biopsychosocial factors and their neural circuitry to enhance and maintain their strength despite allostatic load and prepare for any further adversity in the future (potential or actual). These systems are then maintained to anticipate and respond accordingly to future adversity, and in the event that adversity occurs, the process is recommenced.

Resilience clearly has neurobiological, cognitive-behavioural, emotion regulatory, social and physical underpinnings (see Fig. 6)^7,13,70^. Hence, it has been suggested that gaining understanding of the relationship between neurobiological and social development during adolescence may reveal its underpinnings^71^. However, how neurobiological and psychosocial factors influence each other in adolescence to produce resilience is not well understood. One possibility, besides the operation of plasticity genes, is the involvement of the cortico-limbic circuitry. The cortico-limbic circuitry is involved in the regulation of many processes such as stress (e.g. the HPA axis, the autonomic nervous system (ANS) and the immune system (pro- and anti-inflammatory cytokines), neural growth (e.g. brain derived neurotrophic factor (BDNF), emotion and cognitive processing, social behaviour (e.g. neuropeptides (oxytocin (OXT) and arginine vasopressin (AVP)). This is extremely complicated, so we discuss only key neurobiological and psychosocial determinants that influence each other and potentially contribute to produce resilience in adolescents. Ultimately, it is from these systems that resilience emerges.

### Neurocircuitry of resilience

Structural and functional brain circuitries involved in emotion, stress and behavioural regulation as well as cognitive processes and social behaviour have been identified as the most important in the development and maintenance of resilience, with efficient processing and regulation as indicators^72,73^. These brain circuitries include, in particular, the cortico-limbic regions^66^. From the perspective of brain structural connectivity, the anterior corpus callosum (CC), which projects to anterior cortical regions that are involved in cognitive processes and the reappraisal of negative information, also seems to be implicated in resilience in adolescents^74,75^. Resting-state functional connectivity (rsFC) analysis of associations also shows a heightened rsFC between the anterior default mode network (DMN), the right central executive network (CEN) and ‘trait’ resilience in adolescents^76^.

Augmentation of the amygdala-Prefrontal Cortex (PFC) coupling has been identified as a neural mechanism of adaptation, countering increased amygdala reactivity to adversity in older adolescents^77^. This study did not assess the impact of early to middle adolescence adversity, but it nonetheless suggests that the timing of the adversity may have a significant impact on resilience. On the other hand, in a separate study, stronger connectivity between the hippocampus and the ventromedial PFC (vmPFC) during aversive learning—prospectively predicted improvements in anxiety symptoms as an index of recovery from prior institutionalisation, considered to be a significant form of early adversity in adolescents^78^. Overall, these studies, whether structural, functional or coupled with connectivity, show that resilience emerges from the engagement of the corticolimbic regions involved in stress, emotion and behavioural regulation, social behaviours as well as cognitive functions.

Interestingly, the hippocampus may be emerging as an important brain region for adversity-predicated resilience. In healthy young adults with high resilience despite high adversity (measured with the Resilience Scale (RS), a measure of personal competence and acceptance^79^), improved functional coupling between the ventral striatum (VS), ventral tegmental area (VTA) and the hippocampus, as well as increased activity of the VTA and hippocampus were observed, suggesting a possible protective mechanism^80^. In our own data, we have observed negative associations between left hippocampal volumes and intrinsic resilience (measured with BRS^37^) in healthy mid-adolescent girls (aged 14–15 years) who had been exposed to higher levels of emotional trauma, suggestive of intensified synaptic pruning at this developmental stage^81^. Therefore, it is likely that the hippocampus is involved in various resilience-related processes, such as information processing, stress and emotion regulation (ER).

### Psychosocial components of resilience

A constellation of psychosocial factors that span cognitive, behavioural and existential domains to index resilience in response to stress or trauma have been identified^4,82^. These include cognitive flexibility, ER, active coping skills, maintenance of supportive social networks, upholding a personal moral compass and enhancing physical well-being. These factors are influenced by, and in turn, influence neurobiology. Social influence, which takes many forms, but at its core is the giving (analogous to altruism) and receiving of social support, particularly to and from peers and family, plays a significant role in protecting from the negative effects of adversity^83^.

Recent evidence suggests that the giving of support may be more beneficial than the receiving of support as it reduces physiological responses to stress (blood pressure (BP) and salivary alpha-amylase secretion) in experimental studies^84^, reduces stress-related activity in cortico-limbic regions and increases activity in reward processing regions^85^. On the other hand, the receiving of support provides security and safety via activation of cortical regions that potentially inhibit the sympathetic nervous system (SNS) and inflammatory processes^86^. Therefore, the giving and receiving (perceived or actual) of social support have significant implications for health and resilience^86^^,87^.

These effects have not been assessed in adolescents who are more likely to be experiencing heightened neurobiological sensitivity to social contexts^60,83^. This is particularly important in socially adverse threat and deprivation contexts. Indeed, these stressors may activate social transduction pathways that upregulate inflammatory activity and sensitise to further social-environmental adversity^88^. Social transduction pathways include the HPA axis and the SNS, the salience network in the brain and associated neurocognitive and affective processes involved in the appraisal of social threat^88,89^, to influence behaviours.

### Emotion-focused indicators of resilience

Strong ER skills are considered to be fundamental to resilience, and ER strategies such as cognitive reappraisal are associated with other psychosocial indices of resilience^90,91^. Cognitive reappraisal is the ability to monitor and assess thoughts and replace negative thoughts with positive ones, and it involves conscious reassessment or reinterpretation of adversity to find a positive perspective^4^, and therefore regulate one’s emotions. At the neural level, in both adults and adolescents, emotion regulatory regions include the cortico-limbic network^92^ and parieto-temporal regions^93,94^. Indeed, reappraisal strategies affected by emotional content have been observed to be more effective than distraction in diminishing negative affect in adolescents^95^, and positive reappraisal is a significant predictor of adolescent-perceived resilience^90^. However, such studies have not been conducted in the context of adversity in adolescents.

### Cognitive indices of resilience

The adaptation-based approach suggests that executive functions such as attention, problem-solving and decision-making as well as learning and memory processes are enhanced by some types of adversity and contribute to reflect greater resilience^96^. These enhanced cognitive abilities are the most efficient means of overcoming adversity—allowing the individual to survive intact and continue functioning at an optimal level. This has evolutionary implications. If a species cannot maintain contextual vigilance, fast and critical appraisal of situations, strategic problem-solving and be decisive after prior exposure to a threat, its survival will be short-lived. However, this area is grossly under-researched, and as such it is not clear which of these cognitive abilities and derivatives are most indicative of resilience, particularly in adolescents.

### Biological systems

#### Neuroendocrine system and neuropeptides

The primary neuroendocrine system serving as an interface between the central nervous system (particularly the brain) and the peripheral endocrine systems is the HPA axis, which is most responsive to stress. Stress exposure dysregulates the HPA axis but resilience-enhancing activities and strategies, e.g. developmental social buffering^97^, with parental support in children and peer support in adolescents, protect against this dysregulation^98^. The exact mechanisms for this are not understood, but may involve OXT effects^99^, cortico-limbic network effects^100,101^ and the actions of BDNF^102^. BDNF is a neurotrophic factor involved in the growth, differentiation, maintenance and survival of neurons^103^. Indeed, animal evidence reveals that adulthood affiliative maternal and peer interactions are facilitated by high levels of BDNF in corticolimbic regions including the hippocampus, frontal cortex and hypothalamus, but only peer affiliations are independently enhanced by increased OXT receptor levels in the amygdala^102^.

The evidence of such mechanisms in adolescents is sorely lacking as pointed out by Holstinar and colleagues^104^, and indeed, amplification of the cortisol response and attenuation of OXT rather than buffering effects have also been observed in adolescents^105^. Therefore, OXT with its dynamic interplay with AVP is involved in social behaviours, social and emotional memory and recognition, cognition, attachment and tolerance development^106–108^, in a context-dependent manner^109^, via its anti-inflammatory effects^110^. Importantly, the OXT system has been identified as playing a central role in biobehavioural synchrony, which is the coordination of neurobiological and social processes for social growth and development, and a sensitive-period perspective on resilience, when neuroplasticity is highest, is crucial for the assessment of this synchrony^111^.

#### Immune system factors

It is increasingly recognised that stress/adversity stimulates the release of proinflammatory cytokines from microglia and increases neuroinflammation in stress-sensitive brain regions, altering their structure and function^112,113^. However, suppression of pro-inflammatory cytokines may be markers of resilience^112,114,115^, and heightened peripheral inflammation may be a predictor or consequence of resilience to stress, perhaps because inflammatory markers influence behavioural outcomes via HPA axis regulation and hippocampal neurogenesis^116^. Although attenuation of inflammation seems to be linked to resilience from adversity^117,118^, this line of investigation is still in its infancy.

#### Neurotrophic factors

BDNF genes are highly expressed in stress-sensitive corticolimbic brain regions involved in emotion and cognitive processing, e.g. PFC, hippocampus and amygdala^119^, hence their involvement in stress regulation (as discussed above). The evidence for a critical role of BDNF in resilience during development is largely based on animal models of chronic stress^120–122^, which significantly implicate hippocampal BDNF mediation^120^. BDNF genes are also involved in the development of neural circuits that control coping mechanisms^123^, and human intervention studies also show that psychosocial factors such as the brain friendly trio (BFT), comprising exercise, dietary energy restriction and cognitive stimulation, all upregulate BDNF in the corticolimbic regions to optimise brain health and resistance to disease^124,125^. Furthermore, implementation of BFT, both before and after adversity induces BDNF expression in corticolimbic brain regions, where it protects neurons from damage by modulating synaptic plasticity and controlling neurogenesis. Finally, BDNF is also involved in social behaviours as demonstrated by animal models of social behaviours^102^.

## Proposed integrated resilience model

Our proposed model positions adversity at the core of processes that lead to resilience. Adversity exerts ‘pressure’ on the neural integrity and function of stress-responsive systems and affiliated processes. We posit that without such ‘testing’, resilience cannot fully assume its proper form. In this model, resilience consists of a number of skills that are either newly acquired or have been previously acquired but are then adapted and transferred for deployment in new contexts. These skills apply across neural, biological, cognitive, emotional and social domains. It is important to note that we frame this model within adolescence because this is the optimal developmental period in which research can elucidate how resilience emerges and develops. Therefore, our model of adversity-driven resilience is dynamic, and it incorporates its neurobiological and psychosocial determinants. It allows for a number of potential trajectories that are determined in part by features of adversity. In addition to informing research, our model provides a framework for integration of its clinical components aimed at enhancing resilience in practice.

In the model, resilience ‘traits’, referred to as intrinsic factors, such as genetic and personality factors, are thought to be sufficient for individuals to respond effectively to adversity of low intensity or short duration. As such, intrinsic resilience operates both in and outside of adversity (see Fig. 6). For instance, those low in neuroticism, but high in agreeableness, extraversion, openness and conscientiousness while carrying the protective allelic variants of plasticity genes, are more likely to withstand mild forms of adversity.

However, intrinsic factors alone may not be sufficient to withstand moderate to severe (intense or prolonged) types of adversity. In these instances, additional resources are required to cope with current stressors and also temper and fortify against future adversity. This is when promotive psychosocial factors that come to the fore from childhood to adolescence such as healthy lifestyles and supportive environments build resilience, again within, and outside, the context of adversity^126^. For instance, harnessing positive emotions in daily life^127^, engagement in positive activities and behaviours, e.g. physical activity/exercise, adequate sleep and nutrition, as well as prosocial behaviours^53^, socially supportive environments and cultivating a sense of belonging and mastery over one’s own environment^128^ may all be key to building resilience. These are some of the psychosocial factors that potentially contribute to resilience building. However, if the adversities encountered by an individual are excessive, then intrinsic and built resilience may be insufficient to maintain adequate functioning in the face of increasing allostatic load.

### Impact of increasing allostatic load

Situations or occasions in which allostatic load is excessive often lead to impairment in stress-responsive systems and their neural underpinnings. In these instances, it may not be possible to maintain intrinsic and built resilience, or, if they are maintained, they may not be able to withstand the ‘pressure’ exerted by adversity-driven allostatic load, especially if they are chronic. This can result in variable impairment of stress-responsive systems, from minimal to significant. Minimal impairment may, for example, manifest as overt maladaptive behaviours, while moderate to significant impairment may manifest as either subthreshold symptomatology or frank psychopathology. It is important to note, however, that the emergence of psychopathology does not preclude the individual from being resilient. This is contingent on the individual demonstrating competence across multiple domains^5^. And so, although adversity can be compromising, it is the ability to overcome this limitation that constitutes resilience. Importantly, stress-responsive systems are adaptive and when their neural underpinnings are moderately to significantly burdened, the initial and logical response is to redistribute allocation of existing and additional resources required to alleviate this burden. In these instances, pre-existing skills that have been successfully deployed in other situations can be considered and applied to novel contexts (*tempering*) to overcome the new adversity. These effects can be bolstered by further acquiring new skills (*fortification*). It is during this period of reallocation and reconfiguration of skills and garnering new ones that both tempered and fortified adaptive resilience emerge and take shape.

Typical strategies to redistribute and gain new skills may include top–down strategies, e.g. cognitive behavioural (CB) techniques^129,130^, ER methods and mindfulness training^129^. Similarly, pharmacotherapy can also contribute to both tempering and fortification from the bottom-up. For example, antidepressants essentially facilitate the actions of neurotransmitters that are already in existence, perhaps via BDNF transduction^131^, but may also, at the perceptual level, modify and normalise aberrant processes such as negative information processing biases^132,133^. This is akin to both tempering and fortification, whereas precursor medications such as tryptophan and nutritional supplementation such as vitamin intake are acting as fortifying agents, as they are additives per se.

Facilitated by their mechanisms, cortico-limbic networks are engaged in repair, modification and strengthening of specific neural structures, functions and connections—essentially enacting tempering and fortification, so that adaptive resilience can be achieved^134–139^. For instance, in adults, mindfulness training and cognitive reappraisal (an ER strategy) seemingly activate similar brain regions, namely the PFC, insula and subcortical amygdala—suggesting actions via a common ‘top-down’ regulatory network^137,140,141^. However, the acquisition of these skills has not been investigated in adolescents, particularly in the context of adversity.

Additionally, stress inoculation, which emerges in response mild to moderate adversity, prepares the individual and systems for future adversity, but also has skill acquisition components that are considered as fortifying. Animal evidence suggests that stress inoculation enhances PFC-dependent cognitive control of behaviours, particularly in situations where flexibility and response inhibition is required^56^, and increases myelination of the medial PFC (mPFC)^55^. It also diminishes stress-induced cortisol elevations^57^ while increasing exploration of novel situations throughout adolescence^142^. However, stress inoculation effects have not been thoroughly assessed in humans, and although stress inoculation therapeutic techniques have been applied in the classroom^143^, evidence in this area remains largely empirical^144^.

Our model captures the dynamic nature of resilience and illustrates the role that the intrinsic components play in its generation. It also shows that studies of ‘trait resilience’ only address one of its components. In our model, a major and important additional component of resilience is a skill that confers adaptive resilience, which cannot, by definition, be fixed. This adaptive component has to be carefully activated by drawing on intrinsic and acquired components of resilience, a process that involves both tempering and fortification occurring iteratively. Whilst being constituted, the intrinsic, building and adaptive components of resilience interact and in doing so, develop further—all the time subject to epigenetic mechanisms^20,33,145^. From this perspective, resilience, as a whole, in response to adversity, is actively invoked by the combination of molecular, hormonal, neural, cognitive, emotional, social and behavioural mechanisms to (1) circumvent the expression of vulnerable phenotypes, (2) prevent the development of vulnerable states, (3) prevent worsening of established psychopathologies and (4) enhance functioning^20^, and at the same time prepare systems to protect them from future adversity.

## Research implications of proposed framework

There needs to be investment on resilience research of adolescents that factors various forms of adversity and the various resilience indicators for the benefit of long-term health and well-being in adulthood. Indeed, animal models of adversity and developmental resilience have directed us with where we should be looking. Furthermore, advanced technology and resources enable novel and sophisticated investigations. This requires identification of tools and targets that are essential for measurement and optimal designs for capturing its dynamic nature.

### Tools

In the first instance, assessment of exposure within a developmental framework is crucial, and the Maltreatment and Abuse Chronology of Exposure (MACE) is the only measure thus far that explicitly considers the timing of adversity from childhood through adolescence^146^. This measure also considers many types of adversities that are not normally assessed in measures such as the Childhood Trauma Questionnaire (CTQ)^147^, mainly peer bullying and witnessing of violence. Therefore, with the MACE, both the type and timing of exposure can be determined to separate childhood from adolescent adversity and assess their independent and combined effects.

Having established the timing and type of adversity, measurement of resilience becomes crucial. Although multiple measures of trait related resilience have been developed and used in most resilience research^148,149^, as well as measurement of plasticity genes and personality factors, our model indicates that these measurements address just one component of resilience, whose measurement in the context of adversity is not satisfactory. However, in conjunction with other indices of resilience they may be useful. Importantly, modification of the genome via epigenetic mechanisms will be particularly useful as it will reveal how genes are affected by changes in environment and experience. Furthermore, as positive and negative environments change the epigenome via epigenetic mechanisms^33,34^, it is possible to assess these changes from blood and salivary sampling^150^. Although this will not be specific to any processes such as tempering or fortification, it will, however, reveal how the adaptive process impacts on gene expression. This area of research, in the context of adversity-predicated resilience is in its infancy.

So far, the component of resilience that reflects adaptation is in fact individual functioning. But functioning cannot be assessed on only one domain, as we have shown that it is multifaceted, with neurobiological and psychosocial factors. Although there is substantial evidence of the psychosocial factors, often measured by subjective rating scales, subtle changes in neurobiology that may reflect or influence these psychosocial effects also need to be factored in and, when combined, these provide potential targets for intervention. Therefore, a composite index of resilience that combines the assessed resilience indicators as initially described and used in a study of maltreated and non-maltreated children^151^ and rural adolescents^152^ will be useful. Composite scores have also been used to assess cognitive performance across multiple domains and neuroimaging in old age psychiatry^153^, and offer useful possibilities for cross-sectional and longitudinal assessment across neurobiological and psychosocial domains. Higher composite index scores reflect greater resilience. It is therefore important that the assessed domains are explicitly stated especially as each domain may require a different indexing system, where the unit of measurement may vary from ordinal, nominal to interval. Total resilience indices may be obtained by the summation of each assessed domain score.

### Targets

These neurobiological and psychosocial indices, where resilience is built, include brain structure, function and connectivity, cognitive functioning (e.g. problems-solving, decision-making, attention and memory processes), assessment of stress responsivity (e.g. HPA axis, neuropeptides, inflammatory markers and neurotrophic factors), ER and processing, coping style and social functioning. Markers of stress responsivity can be reliably assessed from peripheral blood and saliva. Indeed, animal evidence directs us to where we should be looking in this context, with the corticolimbic BDNF DNA methylation a candidate, particularly in the mPFC, hippocampus and amygdala, and detectable in adolescents^33,154–156^. However, although blood BDNF levels may reflect brain tissue levels^157^, the specific corticolimbic regions cannot be easily elucidated in humans. Therefore, changes in structural, functional and connectivity neuroimaging as well as physiology will be useful for detecting subtle resilience changes in functioning to reflect an increase or decrease in resilience^66,72–74,76,93,158–161^. Importantly, measurement of a combination of these factors, rather than individual factors will improve discriminative and predictive power as well as temporal or causal relationships between adversity exposure and resilience^162^.

### A specific example: targeting social sensitivity in adolescents

It has been stated that adolescence is a period characterised by heightened social sensitivity^163,164^, but measurement of this sensitivity has not been systematically performed, particularly in the context of adversity. Although little is known, there is evidence that sensitivity to peer evaluations is heightened among those who have been bullied (threat-related adversity) and have low social status (deprivation-related adversity)^165^ and risk-taking is indeed increased in adolescent girls^166^. Interestingly, moderate social sensitivity is thought to support adaptive decision-making^167^, and possibly greater risk aversion^168^, depending on the social context and whether influenced by peers or parents^169^. Furthermore, neural correlates of moderate social sensitivity and adaptive decision-making have been identified to include the temporo-parietal junction (TPJ,) the insula and the dorsolateral PFC (dlPFC)^167,170^. Additionally, adaptive decision-making and risk-taking are supported by competent ER^171,172^. Therefore, the possibility of prospectively measuring neural and behavioural social sensitivity at multiple timepoints before and after exposure to adversity in different contexts, to establish the emergence and prevalence of adaptive decision-making as a proxy of resilience looks promising.

Furthermore, in the absence of adversity, skills targeting social sensitivity that are learnt as part of development, may reflect the building components of resilience. However, in the presence of adversity and depending on its severity, among those with heightened (or attenuated) sensitivity, low-intensity interventions such as social-emotional learning (SEL), with focus on social skills training and ER^140^, or high-intensity stress-management interventions^173^, e.g. cognitive behavioural therapeutic (CBT) strategies^130^, stress inoculation training^143,144,174^, cognitive training and bias modification^175^ and mindfulness training^141^ may be implemented, and their effects on social sensitivity, decision making and risk taking can be assessed. Such interventions may moderate social sensitivity, decision-making and risk-taking to adaptive levels, and this moderation may reflect both tempered and fortified adaptive resilience. Additionally, experimental studies manipulating social behaviours via administration of intranasal OXT and the use of stressor paradigms (e.g. the Trier Social Stress test, TSST) or Leiden Public Speaking Task (LPST)^176^ and their effects on social sensitivity, decision making, risk taking and HPA axis regulation can reveal anticipatory or preparatory and reactive changes involved in resilience processes, capturing all its components. Evidence from these laboratory-based studies can provide targets for prevention in the real world and treatments in clinical settings. Furthermore, assessment of biological markers such as BDNF and glucocorticoids, including genotyping via saliva and blood sampling may be useful in mapping adversity-predicated changes in social sensitivity. These types of designs would reveal the effects of the intervention or primed/adverse features on neurobiological and psychosocial determinants of resilience. Each assessment measure can then be quantified, and all measures can be summed to produce a composite index of resilience.

### Designs to capture its dynamic nature

Another important factor in elucidating the dynamic nature of resilience is that it cannot be elucidated from one time point or cross-sectional studies. Therefore, prospective longitudinal designs, conducted in the real world, are required that span from pre-adversity to post-adversity, to track the trajectory of functioning across multiple domains. This type of prospective longitudinal design can show the changes in neural structures, functions and affiliated processes from pre-adversity to post-adversity to reflect the emergence and maintenance of resilience. Indeed, maintenance of or delayed emergence of resilience also require testing at multiple time points post-adversity. These prospective studies have implications for prevention and can be conducted in home, school and community environments.

To supplement these real-world prospective studies, there are mechanisms that remain poorly understood, which require testing in controlled environments. For instance, although endogenous peripheral OXT is not considered to be a reliable marker, the exogenous (intranasal) administration that allows for experimental assessment of its causal mechanisms on behavioural change has proven to be useful^177^. This can be performed in laboratory settings, where similar studies can also be performed. Furthermore, the adaptation-based approach which can be followed in a laboratory setting seems to take into account many factors, including the type of adversity exposure, as well as the intervening neurobiological and cognitive mechanisms mediating the development of adaptive skills^44,96^. These laboratory-based studies would require either cross-over, double-blind, randomised-controlled studies, performed pre- and post-intervention, or experimental studies conducted under primed/adverse and unprimed/benign or normal conditions to enhance ecological validity. Interventions targeting resilience following minimal to moderate intensity adversity could include SEL programs^178,179^, the brain friendly trio that includes physical activity^180,181^, nutritional interventions^182^ although energy restriction interventions may be problematic for adolescents, and cognitive training strategies^183^. However, as adversity increases, intense stress-management interventions such as stress inoculation training^143^, CBT strategies^130^ and mindfulness training^141^ may be required.

## Clinical implications of resilience

Fostering resilience is critical to promotion of health, prevention of mental health problems and many other related conditions and their treatment, where it may also reflect recovery^1^. Understanding its mechanisms can only add significant value to these health promotion and mental illness prevention efforts. Indeed, psychosocial strategies such as SEL, including social skills training, can be implemented in the presence or absence of adversity exposure to aid in building and tempering stress-responsive systems against the deleterious effects of future actual and potential adversity and can do so via neuroplasticity through thought and experience. These parallel health promotion and universal prevention of psychopathology. In the presence of adversity exposure, additional adaptation enhancing strategies such as stress inoculation training, CBT, cognitive bias modification, mindfulness strategies and pharmacotherapy to target neural systems involved in emotion and stress regulation, cognitive processes and social behaviours are required. These strategies ultimately effect changes in the neurobiological systems that drive behaviours. These strategies can be stepped up to reflect targeted/indicated prevention, early intervention and treatment of psychopathology. Therefore, in response to various adversities, neurobiological factors will influence resilience via psychosocial effects and vice versa, psychosocial factors will influence resilience through effects on neurobiology^184^. This relationship is depicted in the proposed model. Hence, reliable measurement of resilience as adversity-driven and multifaceted will be crucial for prevention and will provide other targets for interventions to track their effectiveness.

## Conclusions

In this review, we have proposed a novel model to provide understanding of resilience based on adversity and its development in adolescents. This model captures its dynamic nature and specifically focuses on how it emerges during adolescence, and how neurobiological and psychosocial factors influence each other to build and strengthen resilience. Furthermore, it also addresses the individual differences observed in resilience.

This model can guide health promotion, prevention, early intervention and treatment. We believe that resilience does not simply emerge following adversity but is the culmination of a combination of intrinsic factors and experiential learning and adaptation over time, which eventuates in tempering and fortification of stress-responsive systems, and related cognitive, emotional and social processes and the neurocircuitry that subserve these systems and processes. Therefore, it is through experiential acquisition of skills in response to adversity and the impact of these on intrinsic factors through epigenetic mechanisms that shape neurobiological and psychosocial factors that ultimately give rise to resilience. Finally, understanding the underlying mechanisms of resilience as predicated by adversity in adolescents is a necessary endeavour for developing successful targeted interventions for those at increased risk. It is likely to be of immense benefit and aid in preventing a multitude of health, social and behavioural problems.
